# Peritoneal Mesothelioma with Residential Asbestos Exposure. Report of a Case with Long Survival (Seventeen Years) Analyzed by Cgh-Array

**DOI:** 10.3390/ijms18081818

**Published:** 2017-08-22

**Authors:** Gabriella Serio, Federica Pezzuto, Andrea Marzullo, Anna Scattone, Domenica Cavone, Alessandra Punzi, Francesco Fortarezza, Mattia Gentile, Antonia Lucia Buonadonna, Mattia Barbareschi, Luigi Vimercati

**Affiliations:** 1 Department of Emergency and Organ Transplantation, Division of Pathology, Medical School, University of Bari, 11 G. Cesare Square, 70124 Bari, Italy; pezzuto.federica@libero.it (F.P.); andrea.marzullo@uniba.it (A.M.); alessandra.punzi@hotmail.it (A.P.); francescofortarezza.md@gmail.com (F.F.); 2 Division of Pathology, IRCCS, National Cancer Institute “Giovanni Paolo II”, 70124 Bari, Italy; a.scattoneanatopat@libero.it; 3 Department of Interdisciplinary Medicine, Occupational Health Division, Medical School, University of Bari, 70124 Bari, Italy; domenica.cavone@uniba.it (D.C.); luigi.vimercati@uniba.it (L.V.); 4 Division of Medical Genetics “Di Venere Hospital”, 70124 Bari, Italy; mattiagentile@libero.it (M.G.); albuonadonna@libero.it (A.L.B.); 5 Department of Pathology, Santa Chiara Hospital, 38121 Trento, Italy; mattiabarbareschi@apss.tn.it

**Keywords:** peritoneum, mesothelioma, molecular-array, asbestos

## Abstract

Malignant mesothelioma is a rare and aggressive tumor with limited therapeutic options. We report a case of a malignant peritoneal mesothelioma (MPM) epithelioid type, with environmental asbestos exposure, in a 36-year-old man, with a long survival (17 years). The patient received standard treatment which included cytoreductive surgery (CRS) and hyperthermic intraperitoneal chemotherapy (HIPEC). Methods and Results: Molecular analysis with comparative genomic hybridization (CGH)-array was performed on paraffin-embedded tumoral samples. Multiple chromosomal imbalances were detected. The gains were prevalent. Losses at 1q21, 2q11.1→q13, 8p23.1, 9p12→p11, 9q21.33→q33.1, 9q12→q21.33, and 17p12→p11.2 are observed. Chromosome band 3p21 (*BAP1*), 9p21 (*CDKN2A*) and 22q12 (*NF2*) are not affected. *Conclusions:* the defects observed in this case are uncommon in malignant peritoneal mesothelioma. Some chromosomal aberrations that appear to be random here, might actually be relevant events explaining the response to therapy, the long survival and, finally, may be considered useful prognostic factors in peritoneal malignant mesothelioma (PMM).

## 1. Introduction

Peritoneal malignant mesothelioma (PMM) is a rare, lethal malignancy, whose main known cause is occupational or environmental asbestos exposure. Other possible risk factors are radiation exposure and genetic predisposition [1]. The peritoneal mesothelioma incidence is rising continuously without peaking; often the disease is unresectable and the patient’s prognosis poor (10–13 months) [2]. In recent years, the prognosis has improved due to therapeutic advancements such as surgery in combination with other treatment modalities such as hyperthermic intraperitoneal chemotherapy or radiation [3]. It has been ascertained that mesothelioma generally occurs in elderly asbestos-exposed men. When the tumor occurs in younger people, generally a genetic predisposition and environmental exposure to asbestos or other mineral fibers are implicated. Genetic susceptibility alone cannot explain mesothelioma in young people, but an inordinate sensitivity to these fibers must be considered. In fact, in familial mesothelioma, germline bands 3p21 (*BAP1*) mutations make the members susceptible to the development of cancer even in the presence of low amounts of inhaled asbestos [4]. 

Patients younger than 40 years appear to have a significantly longer survival compared to older patients (both in pleural and peritoneal mesothelioma). In a recent report, Thomas et al. [5] suggested that female gender, peritoneal site, histology, surgery, and radiation therapy are the better prognostic factors. However, the authors emphasize the need for genetic study of the tumor to identify recurring changes that can define the disease at a young age. Therefore, the identification of genetic changes will produce the development of new agents targeting these oncogenic abnormalities. Many DNA copy number alterations (CNAs) have been identified in peritoneal malignant mesothelioma [6]. Generally, the genetic alterations observed in malignant mesothelioma (MM) are deletions in 1p21-22, 1p36, 3p21, 4p, 4p12-13, 4q, 4q31-32, 6q14-25, 6q22, 9p21, 10p13-pter, 13q13-14, 14q, 14q12-24, 14q32, 15q15, 17p12-13, 17p12-pter, and 22q12, and amplifications in 1q23, 1q32, 1p36.33, 5p, 5p15.1-pter, 7p, 7p14-15, 7q21, 7q31, 8q, 8q22-23, 8q24, and 15q22-25 shown by comparative genomic hybridization (CGH) analysis [7,8,9,10,11,12,13,14,15]. Deletion of 9p21 is particularly frequent in MM, especially in the biphasic histotype, although this deletion has been reported to be less frequent in peritoneal than in pleura mesothelioma [15,16,17]. Also 3p21 and 22q12 losses are less common in peritoneal mesothelioma. Generally, a higher frequency of loss was seen in pleural MM, gains were seen more frequently in peritoneal MM, including regions at 3q, 7q, 8p, 9p, 16p, and 20q [16,18,19]. 

This study describes the genetic alterations observed in a case of diffuse asbestos-related peritoneal mesothelioma affecting a young man (under 40 years) who has survived and is currently disease-free.

## 2. Results

**Figure 1:**
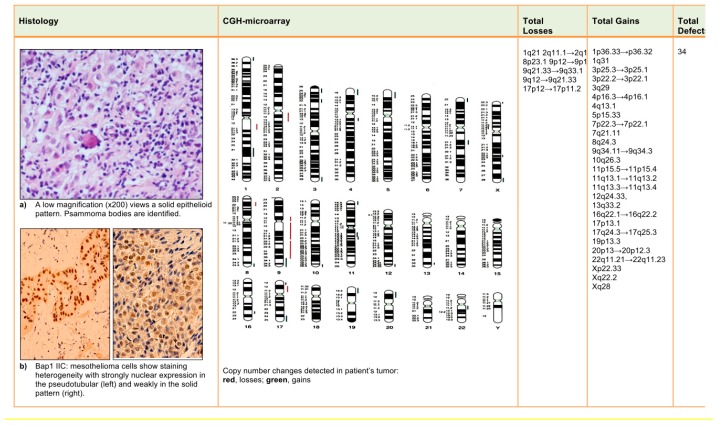
Malignant peritoneal mesothelioma: histology, immunohistochemical (IHC) and comparative genomic hybridization (CGH)-array results.

The CGH-array analysis revealed multiple chromosomal abnormalities (27 gains and 7 losses). Deletions were found at 1q21, 2q11.1→2q13, 8p23.1, 9p12→9p11, 9q21.33→9q33.1, 9q12→9q21.33, and 17p12→17p11.2. Amplifications were at 1p36.33→1p36.32, 1q31, 3p25.3→3p25.1, 3p22.2→3p22.1, 3q29, 4p16.3→4p16.1, 4q13.1, 5p15.33, 7p22.3→7p22.1, 7q21.11, 8q24.3, 9q34.11→9q34.3, 10q26.3, 11p15.5→11p15.4, 11q13.1→11q13.2, 11q13.3→11q13.4, 12q24.33, 13q33.2, 16q22.1→16q22.2, 17p13.1, 17q24.3→17q25.3, 19p13.3, 20p13→20p12.3, 22q11.21→22q11.23, Xp22.33, Xq22.2, and Xq28. CNAs detected are shown in Figure 1. 

## 3. Discussion

Epidemiological studies report a significant risk of mesothelioma in people exposed to asbestos in non-occupational settings. The prominent characteristic of non-occupational exposure is the considerably younger age at the start of exposure, and hence the risk of a longer exposure and latency. Furthermore, the risk of MM is increased with cumulative dose of asbestos even in analyses limited to non-occupationally exposed subjects [20]. Peritoneal mesothelioma is difficult to diagnose, particularly when the tumor affects women and younger patients. Currently, cytoreductive surgery, combined with hyperthermic intraperitoneal chemoperfusion is the best treatment for young patients. However, clinical trials have shown that this treatment improves median overall survival by 27–46 months [21]. In a multivariate analysis, Thomas et al. [5] report that younger age was a favorable independent prognostic factor, including after multimodal treatments. Because multimodal treatment is associated with a significant perioperative risk it is necessary to consider prognostic markers during patient selection. At present, the morphological growth patterns of the tumor and the mitotic index are the prevalent aspects used to select patient groups to be submitted to treatment options [21,22]. 

Frequent molecular alterations [deletion/mutation] at *CDKN2A* (9p21), *NF2* (22q12), and *BAP1* (3p21) are considered the predictive prognostic factors for progression-free survival and overall survival in malignant peritoneal mesothelioma (MPM) [23]. The molecular pathway and mechanism still remains unknown due to a lack of large-scale studies. Currently, in oncology, these alterations are not decisive, nor do they affect the choice of chemotherapy. In a younger patient with non-asbestos-related peritoneal mesothelioma, epithelioid type, Sheffield et al. [16] observed that loss of function in *NF2* was correlated with a worse prognosis and no response to chemotherapy. By contrast, a middle-aged woman with non-asbestos-related peritoneal mesothelioma, sarcomatoid type, with mutations in *NF2* (22q12.1), *CDKN2A* (9p21), *p53* (17p12), and *LATS2* (13q12), had an excellent response to treatment. Hence the question, whether it is appropriate to exclude patients with sarcomatoid mesothelioma from aggressive treatment options. *BAP1* was normal in both cases. 

Alakus et al. [24], did not observe *CDKN2A* changes when analyzing a series of peritoneal mesothelioma. *BAP1* was lost or inactivated in three of seven cases of peritoneal mesothelioma, suggesting a potential genetic predisposition in these patients. The same results were reported by Borczuk et al. [18] in a series of 32 peritoneal mesothelioma cases. However, when considering *BAP1* in other tumors, its loss is a marker of poor prognosis; in mesotheliomas, the real biologic role of BAP1 (germline or somatic mutations) is unclear and it appears to work with different results [25].

Singhi et al. [26], in a recent report of 86 malignant peritoneal mesothelioma, had observed that the absence of *BAP1* nuclear expression correlated with increased mean age and with the epithelioid subtype; the loss or absence of nuclear *BAP1* expression was not associated to asbestos exposure, incomplete cytoreduction, invasion, or metastasis. *BAP1* immunohistochemical staining, loss vs. preserved, was not associated with clinical outcome, whereas the *CDKN2A* and *NF2* deletions were negative prognostic markers.

Our mesothelioma asbestos-related (environmental exposure), epithelioid subtype, shows a high nuclear immunohistochemical *BAP1* expression, the chromosome bands 3p21 (*BAP1*), 9p21 (*CDKN2A*), and 22q12 (*NF2*) are not altered, and the young patient is disease-free.

The Pin2/TRF1-interacting telomerase inhibitor (*PinX1*) is a novel cloned gene located at human chromosome 8p23, playing a vital role in maintaining telomeres length and chromosome stability [27]. Loss of heterozygosity (LOH) regions, *PinX1* overexpression or inhibition are detected in human malignancies. Thus, *PinX1* might be considered as a putative tumor suppressor gene. The mechanism for *PinX1*-gene inactivation in human cancer is not clear. Recently, it has been demonstrated that *PinX1* expression is directly activated by *p53* in cervical cancer. The authors suggest that *PinX1* inhibition via *p53* transcriptional activity results in the enhancement of telomerase activity [28]. *PinX1* has also been demonstrated to be a new potential cancer therapy target (i.e., fibrosarcoma, hepatocellular carcinoma, breast cancer, gastric carcinoma, lymphoma, etc.). Interestingly, our case presented loss at 8p23. 

Gelsolin, a Ca^2+^-regulated actin filament severing, capping, and nucleating protein, is a ubiquitous, multifunctional regulator of cell structure and metabolism. Gelsolin (*GSN*) can act as a transcriptional cofactor in signal transduction and its own expression and function can be influenced by epigenetic changes. In humans, intracellular (*cGSN*) and extracellular forms (*pGSN*) of *GSN* are encoded by genes on chromosome 9q33. A decreased expression of *cGSN* has been observed in breast, bladder, lung, colorectal, ovarian cancers, etc. and was associated with cell proliferation and survival [29]. *GSN* is involved in the modulation of several signaling pathways, including c-erb-2/EGFR, *p53*, PI3K, Ras-PI3K-Rac, etc. [27,30]. *GSN* represses transactivation of *p53* via inhibition of nuclear translocation of p53, thus inhibiting *p53*-mediated apoptosis in hepatocarcinoma cells [31]. Our case reports 9q33 loss, being the putative site of *GSN*, this tumor suppressor gene.

Finally, Telomerase is a ribonucleoprotein complex mainly composed of a reverse transcriptase catalytic subunit which copies a template region of its RNA subunit to the end of the telomere. Human telomeres function as a protective structure capping the ends of chromosomes. Dysfunction of telomeres plays an important role in cancer initiation and progression. The active human telomerase enzyme is composed of human telomerase reverse transcriptase (*hTERT*), encoded by the TERT gene located at 5p15.33; human telomerase (*hTR*) encoded by the TERC gene at 3q26.3; and dyskerin encoded by the *DKC1* gene located at Xq28. Gains at 5p15.33, 3q26.3, and Xq28 are the regions known to be involved in cancer cells [18,32,33]. Gains at 5p15.33 and Xq38 are shown in our case. So, detection of TERT amplification may be useful to reveal an initial mesothelioma but we should also consider that TERT promoter mutations might be associated with inactivation of many cell cycle regulator genes.

Our study is coherent with the CGH findings reported in the literature for the mesothelioma diagnosis, but failed to identify a compelling alteration to explain the patient’s outcomes. So, CGH-array may be a helpful and sometimes definitive approach in the diagnosis of mesothelioma, to evidence the role of TSGs in the tumor pathogenesis, but it is not the method to detect genes-status involved in the tumor progression. Therefore, whole-genome sequencing data represent a crucial milestone not only in the understanding of the mesothelioma pathogenesis, but also to detect the genes that are important for personalized therapy. Next-generation sequencing (NGS) coupled with bioinformatics analysis of the somatic mutations would allow for the screening of tumor-specific mutated proteins, candidate targets for the design of individualized therapy. Our patient, who received a combined chemotherapy with raltitrexed and oxaliplatin, appears to have responded to therapy not merely by chance. The combination of ralitrexed and oxaliplatin would have a synergistic effect on the early inhibition of DNA synthesis promoted by the mutations described [34]. No alteration of the chromosome 18p11.32 (site of the thymidylate synthase gene) was observed in our case.

A smaller total number of losses in the tumoral chromosomes might be related with a longer survival [7,11], but further investigations need to be performed to characterize the recurrent mutations useful to improve the prognosis.

## 4. Material and Methods

### 4.1. Case Report: Clinical History an Pathological Features

In May 1999, a 36-year-old man was admitted to the Hospital “Azienda Ospedaliera Universitaria Policlinico” in Bari, Apulia Region—Southern Italy, for ascites, abdominal pain, and weight loss. His past medical history was unremarkable; he had been a smoker (20 cigarettes per day) since he was 13 years old. A CT-scan revealed that there was irregular thickening (thickness > 2 mm) of the peritoneum and suspect nodules but no abdominal viscera invasion. Routine laboratory tests were unremarkable. Tumor markers were negative. An exploratory laparotomy with biopsy was performed. Histology showed MM with a solid epithelial (60%, predominant subtype) (Figure 1a) and pseudotubular (40%, subdominant) pattern. The neoplastic cells were generally polygonal, cuboidal or low columnar, with a pale-to-eosinophilic, abundant cytoplasm; cellular atypia ranged from mild to moderate. Nucleoli were prominent and eosinophilic. Nuclear pseudoinclusions and psammoma bodies were also seen (Figure 1a). The mitotic count was 2/mm^2^. Necrotic foci and vascular invasion were not found. Immunohistochemical (IHC) analysis showed intense expression of 5/6 cytokeratins, calretinin, WT-1, HBME-1, and vimentin. Ki67-index was 5%. In addition, IHC detection of BAP-1 was performed using a primary anti-human *BAP1* antibody (C-4, Santa Cruz Biotechnology, Santa Cruz, CA, USA). Tumor tissue showed a strong/moderate *BAP1* nuclear expression in pseudo-tubular and solid epithelioid pattern (Figure 1b). 

Patient opted for surgery at a specialist center (Institute Gustave Roussy, France); where he underwent cytoreductive surgery (CRS) and hyperthermic intraperitoneal chemotherapy with oxaliplatin (HIPEC). Complete cytoreduction of all macroscopic tumors (>2 mm) was combined with peritonectomy, omentectomy and organ resection (cholecystectomy, half the distal small intestine and right colon). In accordance with the French hospital protocol, preoperatively the patient underwent six cycles of chemotherapy (raltitrexed (Tomudex^®^) and oxaliplatin combined). The patient was enrolled in the Apulia Regional Mesothelioma Register by the pathology department, in compliance with Italian law for the compulsory notification of new cases of MM. According to the standardized mesothelioma register questionnaire to detect asbestos exposure, lifestyle habits and work history, including a possible asbestos exposure during military service, were investigated. The work history of cohabiting family members and a family history of cancer were also evaluated. Exposure during leisure, travel or hobby activities was excluded, as well as any exposure to ionizing radiation. The ascertained asbestos exposure was residential: he had lived near a source of asbestos pollution, in-situ in buildings, at a distance of less than 15 m for 36 years. In fact, the subject had always lived, since birth, in an apartment overlooking military barracks, built in the period 1920–1930, and also played football there. The presence of asbestos (eternit) in the barracks had been ascertained in roofs and chimneys. In 2001, reclaiming of the asbestos roofing began, and ended in 2006. At follow-up in November 2016 (Positron Emission Tomography (PET)/CT-scan performed in July 2016 was negative), more than 17 years after diagnosis, the patient is alive and disease-free without recurrence. Currently, he has chronic diarrhea and chronic abdominal pain. 

### 4.2. CGH-Array Analysis

Informed written consent to the use of histological samples for additional studies was obtained. Molecular analysis with CGH-array was performed on paraffin-embedded tissue. Genomic DNA was extracted from 5-µm sections of paraffin-embedded tissue with the Dneasy Tissue Kit (Qiagen, Hilden, Germany) according to the manufacturer’s instructions. Normal sex-matched DNA was extracted from peripheral blood lymphocytes according to standard hybridization procedures (Nucleon BACC3, Amersham Pharmacia Biotech, Bucks, UK). Array-CGH with a genomic resolution of about 0.5 Mb, increasing to 0.25 Mb in the subtelomeric regions, was carried out using the Cytochip V3 genomeARRAY slide (Techno Genetics Srl-Bouty Spa, Milan, Italy), containing 5.380 BAC clones, according to the manufacturer’s instructions. Slides were scanned at 633 nm (Cy3) and 543 nm (Cy5) using the Scan ArrayGx (PerkinElmer, Waltham, MA, USA). Image analysis was done using BlueFuse software (Bluegnome Limited, Cambridge, UK). Once the positions of the biological sample were known, a powerful quantification algorithm was used to calculate the amount of signal at each spot location. For each clone, a log2 of the ratio Cy3/Cy5 fluorescent intensity was calculated. The raw results delivered by quantification were subjected to a series of post-processing stages including normalization, data exclusion, and identification of copy number change regions considering the replicate standard deviation values, the internal controls, the degree of confidence, and the median of the log_2_ ratio of clones in the regions. Data points lying beyond three standard deviations were considered to be part of a change analysis region. Regions exceeding the ratio thresholds of log 0.3 and log −0.3 and containing at least one clone were considered to be amplifications or deletions, respectively. The results of experiments were visualized on the copy number panel. Full reports, including an ISCN summary of regions of change, were provided as Excel spreadsheets in the results directory. 

## 5. Conclusions

On the basis of the evidence of genomic abnormalities found in our case, related to asbestos exposure in a patient with a supposed genetic cancer predisposition, these may be very important in the choice of therapy, but systematic molecular analyses are needed to understand the complexity and heterogeneity of the chromosomal aberrations that characterize such malignancies. Chromosomal regions of common allelic loss or gain may contain more than one gene associated with clinical-pathological features such as age, histological type, therapy response, or survival. Some chromosomal aberrations that appear to be random here might actually be relevant events explaining the response to therapy, the long survival and, finally, may be considered useful prognostic factors in PMM.
